# After the revolution: how is Cryo-EM contributing to muscle research?

**DOI:** 10.1007/s10974-019-09537-7

**Published:** 2019-07-13

**Authors:** Marston Bradshaw, Danielle M. Paul

**Affiliations:** Department of Physiology & Pharmacology, University of Bristol, Bristol, BS8 1TD UK

**Keywords:** Cryo-EM, Actin, Thin filament, Myosin, 3D reconstruction

## Abstract

The technique of electron microscopy (EM) has been fundamental to muscle research since the days of Huxley and Hanson. Direct observation of how proteins in the sarcomere are arranged and visualising the changes that occur upon activation have greatly increased our understanding of function. In the 1980s specimen preparation techniques for biological EM moved away from traditional fixing and staining. The technique known as cryo-electron microscopy (Cryo-EM) was developed, which involves rapidly freezing proteins in liquid ethane which maintains them in a near native state. Within the last 5 years there has been a step change in the achievable resolution using Cryo-EM. This ‘resolution revolution’ can be attributed to advances in detector technology, microscope automation and maximum likelihood image processing. In this article we look at how Cryo-EM has contributed to the field of muscle research in this post revolution era, focussing on recently published high resolution structures of sarcomeric proteins.

## Introduction

In 2017 the Nobel Prize for Chemistry was given to Jaques Dubochet, Richard Henderson and Joachim Frank for their pioneering work in establishing the technique of cryo-electron microscopy (Cryo-EM) (Cressey and Callaway 2017). This technique can be briefly described as a process of rapidly freezing proteins in liquid ethane, imaging them in an electron microscope and computing three dimensional (3D) maps from the two dimensional (2D) images obtained. The high speed of freezing prevents crystalline ice formation which would perturb the molecular structure. The protein is preserved in a near native state within the amorphous ice. The interaction of electrons with the proteins gives rise to 2D projection images which contain information about the 3D structure. The 3D map is calculated in a similar way to a CT scan in a hospital, using back-projection methods that combine X-rays at known scanning angles. The difference being that frozen proteins are randomly oriented in the electron beam and their relative orientations are not known. Despite the low signal to noise ratio in Cryo-EM micrographs the image processing techniques of classification, averaging and angle assignment allow the relative orientations of the particles to be determined and a 3D map calculated.

Electron microscopy has been fundamental to our understanding of muscle structure and function from the days of Hanson and Huxley (1953). The development of the plunge freezing technique for Cryo-EM (Dubochet et al. 1988) allowed us to move away from traditional staining and fixation methods and their inherent resolution limits. When heavy metal stains are used, only the molecular envelope of the protein is imaged and the size of the stain particles limits the resolution. With uranyl acetate this limit is ~ 20 Å. The resolution limit when imaging frozen hydrated samples is in practical terms set by the pixel size of the image from which the Nyquist–Shannon limit can be calculated. The pixel size of a typical Cryo-EM session today would be between 0.5 and 1 Å which sets a maximum resolution limit of 1–2 Å.

**Fig. 1:**
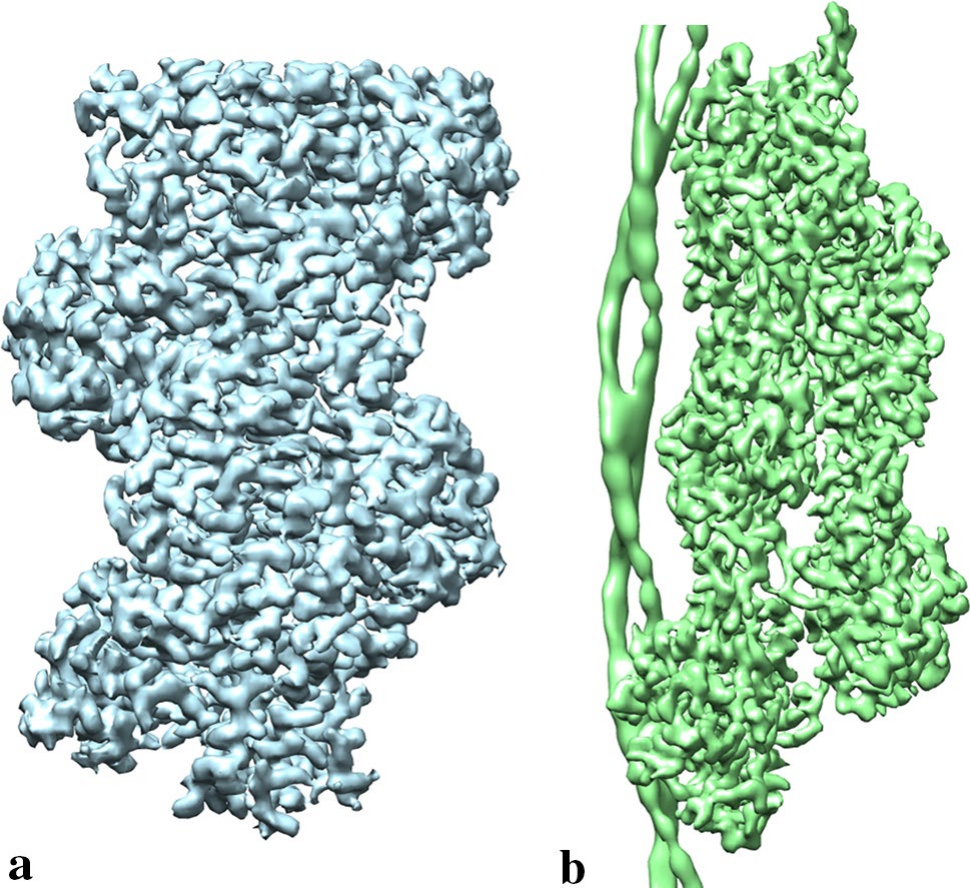
**a** Cryo-EM structure of AMPPNP-actin filaments (EMD-7936, 3.1 Å) (Chou and Pollard 2019). **b** Cryo-EM structure of the F-actin-tropomyosin complex (EMD-6124, 3.7 Å) (von der Ecken et al. 2015). Map comparisons not to scale

This significant development in specimen preparation technique was coupled with the application of statistical approaches to image processing. The use of multi-variate statistical analysis and hierarchical classification established the image processing workflows now known as single particle analysis (SPA) (Frank and van Heel 1982; van Heel and Frank 1981). By grouping sets of similar images of randomly oriented particles together and summing them, the resulting 2D image has increased signal to noise. The relative orientations of all the 2D ‘class averages’ are determined to calculate the 3D reconstruction. It is only recently that the full potential of this technique is being realised, with the Nobel prize being awarded as the pinnacle of what has been referred to as the ‘resolution revolution’ (Kuhlbrandt 2014). In the last 5 years there has been a significant jump in the achievable resolution that the technique can resolve. The highest resolution single particle Cryo-EM map deposited in the Electron Microscopy Data Bank (EMDB) at the time of writing is of apo-ferratin at 1.6 Å (EMDB-9599 & EMDB-0144). This realm of resolution was once something only X-ray crystallographers could hope to achieve. Currently the highest resolution structure of a filamentous muscle protein is F-Actin at 3.1 Å. Two maps of actin have been deposited at this resolution in which you can see side chains and the presence of nucleotide (Chou and Pollard 2019) (Fig. 1a).

This leap in resolution can be attributed to several technological advances the first of which is the use of direct electron detectors in biological EM. These detectors have a vastly improved Detective Quantum Efficiency (DQE), a parameter for specifying the performance of the detector. They also use a rolling shutter mechanism which means micrograph ‘movies’ are collected with ~ 25–40 frames per movie. Recording a movie means that the beam induced movement of the particles can be recorded and subsequently corrected. Large movements of the particles can occur and correcting for that motion is an important first step that improves the resolution of 2D class averages. Automated data acquisition has also become an essential part of current EM methodology, particularly as state-of-the-art microscopes are extremely stable; imaging conditions and alignments do not need adjustment over a typical 2-day data collection session. New camera technology and automation result in large amounts of data being collected, increasing the number of molecular images to be analysed. It is typical that datasets are of the order of hundreds of thousands of particles. Finally, the other major contributing factor is the application of maximum likelihood statistical approaches to image processing. These now well-developed software tools provide objective and reliable criteria for averaging large numbers of particles (Scheres 2012).

In 2014 it was thought that resolution of 2 Å or better would remain the realm of X-ray crystallography (Kuhlbrandt 2014), but within 5 years this has been proved wrong. Achievable resolution is still increasing, but possibly the more exciting outcome is that large numbers of near atomic structures are being published, widening the scope of the biological questions that can be answered. In the next three sections we will look at the impact of Cryo-EM on muscle research and what high-resolution structures of actin, myosin and the sarcomere have revealed about muscle function.

## Cryo-EM structures of F-actin

The first Cryo-EM reconstruction of filamentous actin or F-actin was carried out by Trinick et al. (1986). At roughly 40 Å resolution, the helical substructure and two large domains per actin subunit were observed. At 3.1 Å resolution, the reported resolution level of F-actin structures from Chou and Pollard (2019), detail has dramatically increased, with side chains and nucleotides now visible. The two 3.1 Å maps are of actin with bound AMP.PNP (an ATP analog) and ADP-Pi (ADP with an inorganic phosphate). The first sub 4 Å reconstructions of F-actin in different nucleotide states were reported by Merino et al. (2018). These reconstructions of F-actin established that neither ATP hydrolysis nor phosphate release produce substantial conformational changes. However, when ATP-actin polymerises there are conformational changes in side chains located in the active site which leads to an increase in the rate of ATP hydrolysis. These recent F-actin reconstructions have now matched the level of detail recovered in the X-ray fibre diffraction model from Oda et al. (2009).

## Myosin decorated filaments

Actin based filaments decorated with myosin subfragment-1 (S1) have been a popular object of study as a fully decorated filament exhibits helical symmetry. In 1987, Milligan and Flicker (1987) published Cryo-EM structures of S1 decorated thin filaments and F-actin at roughly 26–30 Å resolution. This work showed the organisation and interactions between the proteins and located tropomyosin as bound to the inner domain of actin. The subnanometer threshold was crossed by Behrmann et al. (2012) with an 8 Å map of the actin-tropomyosin-myosin (ATM) complex in nucleotide free conditions. At this resolution secondary structure elements can be resolved by eye and a pseudoatomic model was calculated. This model defined a large interface between myosin, two actin monomers and a single tropomyosin pseudo repeat, where disease-causing mutations were located.

The 4 Å watershed was passed for a rigor ATM complex by von der Ecken et al. (2016). The average resolution for the map was 3.9 Å, providing further information about the actin myosin interface which appears to be stabilised by hydrophobic interactions. It also showed that binding to actin had little effect on myosin conformation as it was found to be very similar in this complex to rigor-like myosin in the absence of F-actin. Despite the high average resolution of the map, the tropomyosin resolution remained at 7 Å. A similar resolution was achieved for tropomyosin in their earlier analysis of the actin tropomyosin filament (von der Ecken et al. 2015) (Fig. 1b). Despite efforts to refine tropomyosin separately, non-helical regions like the overlap region of the N and C termini were not resolvable.

**Fig. 2:**
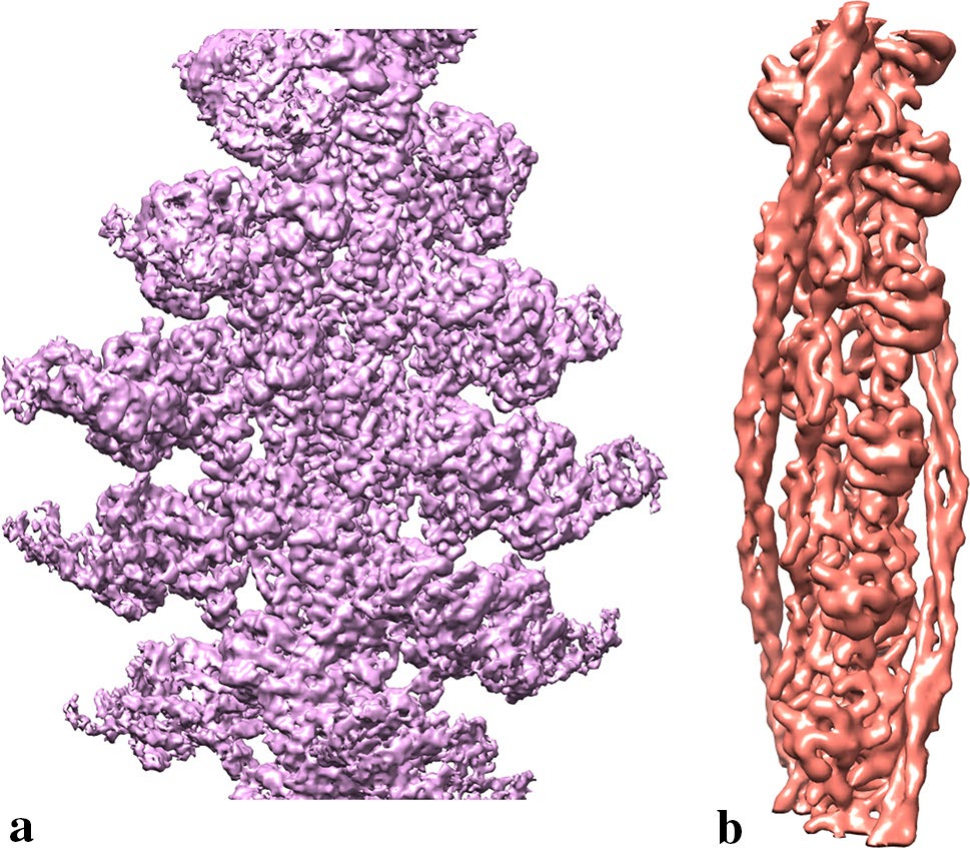
**a** Cryo-EM structure of actin-bound myosin (EMD-7329, 3.2 Å) (Mentes et al. 2018). **b** Cryo-EM structure of native cardiac thin filament with tropomyosin in “closed” state (EMD-3667, 8.0 Å) (Risi et al. 2017). Map comparisons not to scale

Mentes et al. (2018), who studied actomyosin with ADP bound but in the absence of tropomyosin, were able to refine their map to 3.2 Å resolution (Fig. 2a). Their 3D classification revealed two different conformational states of AM.ADP, which is consistent with kinetic studies. When combined with the position resolved in their rigor structure, they were able to put together a sequence of movements corresponding to actin binding and phosphate release. These distinct states were resolved through the application of recently developed protocols for 3D image classification and helical processing (He and Scheres 2017).

## The thin filament

Native thin filaments are mainly composed of F-actin and the regulatory proteins tropomyosin and troponin. The chief component is a helical filament of F-actin. However, the regulatory protein troponin does not lie on the same helical path as actin. The troponin complex (in vertebrate muscle) is known to label every seventh actin monomer and to bind with a 27.5 Å stagger between the two strands of actin. Tropomyosin also cannot be assumed to lie in the same position on every actin subunit, as we have shown in Paul et al. (2017). In the Ca^2+^ bound state tropomyosin was found at a different position on the actin subunit depending on its relative position to troponin. The presence and potential contribution of nebulin should also be considered in native skeletal thin filament preparations.

Since helical averaging techniques have been used for most actin-based filament reconstructions, it is of no surprise that some structural studies of the thin filament have not considered the inherent symmetry mismatch between actin monomers and troponin/tropomyosin. By assuming that the helical symmetry of actin applies to the entire thin filament, not only is any contribution from troponin lost, but the troponin density is averaged over every actin subunit, effectively strengthening other regions of the map (Squire and Morris 1998). The first Cryo-EM reconstruction of the thin filament was helical (Xu et al. 1999) and displayed no discernible density for troponin.

Several attempts have been made to correctly consider the stoichiometry of binding of troponin. The Paul and Lehman groups have developed methods for the analysis of negatively stained thin filament data. Ten different and distinct starting models were used in Yang et al. (2014) and refinement resulted in a consensus position for troponin and a 3D reconstruction at 25 Å. In Paul et al. (2017) we describe two maps of the thin filament in the relaxed and active state (EMDB-3578 and EMDB-3567). Selecting troponin directly, focussed classification and applying a two-strand averaging technique led to 3D reconstructions that were not based on a starting model. Large conformational changes in troponin that occur on the addition of Ca^2+^ were observed and the position of tropomyosin on actin depended on its axial position relative to troponin.

In 2001 Narita et al. (2001) collected a Cryo-EM dataset of thin filament segments prepared at high and low Ca^2+^ conditions. Non-actin density was defined by the subtraction of an atomic F-actin model. This non-actin density was removed and added back onto every seventh monomer before refinement. Troponin density was described, and the map resolution initially reported as 35 Å. The most recent Cryo-EM study of the thin filament was by Risi et al. (2017) at 8 Å resolution. High-quality images of native porcine thin filaments clearly displaying troponin were collected and a helical single particle approach was taken. However, no discernible contribution from troponin could be detected in the 3D reconstruction (Fig. 2b).

## Resolution limits

The actin-based Cryo-EM reconstructions described above have used a helical single particle approach to their image processing. This form of image processing makes use of the helical parameters that actin exhibits in the form of averaging. This increases the signal-to-noise in the data and effectively multiplies the number of particles you have by the number of asymmetric units in the map. A widely used approach is iterative helical real space refinement (IHRSR) (Egelman 2000), which determines the helical symmetry before averaging. The fact that actin monomers sit on a helical path is well recognised. However, deviations from helical symmetry occur, the filamentous nature of actin produces curvature, and actin exhibits lateral slipping, intrinsic disorder and flexibility. When you also consider non-stoichiometrically bound proteins, sparse binding, or partial occupancy of nucleotides, the situation becomes more complicated. Post-translational modifications of actin may also affect its structure and filament formation (Terman and Kashina 2013; Wilson et al. 2016). All of these factors mean that imposing long range helical order can result in loss of detail.

Von der Ecken et al. (2015, 2016) successfully developed and applied hybrid methods that are based on a single particle approach but include helical consistency checks and custom restrains on angular space in refinement. The application of this methodology increased the resolution of the flexible ligand binding sites. These advances in methodology have pushed the resolution to ~ 3 Å, but is there scope to go further? The inherent flexibility of F-actin is a major obstacle and without trying to artificially stabilise the filaments we may need to focus on in silico purification. This would need image classification tools that were sensitive enough to sort heterogenous populations using ligand occupation or sub domain position.

## Myosin filament structure

When helical single particle analysis was applied to images of frozen hydrated tarantula thick filaments in 2005, features in the backbone started to be resolved for the first time (Woodhead et al. 2005). Tarantula thick filaments are known to have heads that lie on a helical path and as such the application of the IHRSR helical averaging techniques was beneficial in the analysis (Egelman 2000). Another observation was that the two heads within one myosin molecule were interacting with each other, something previously seen in a study of smooth muscle myosin heads (Wendt et al. 2001).

**Fig. 3:**
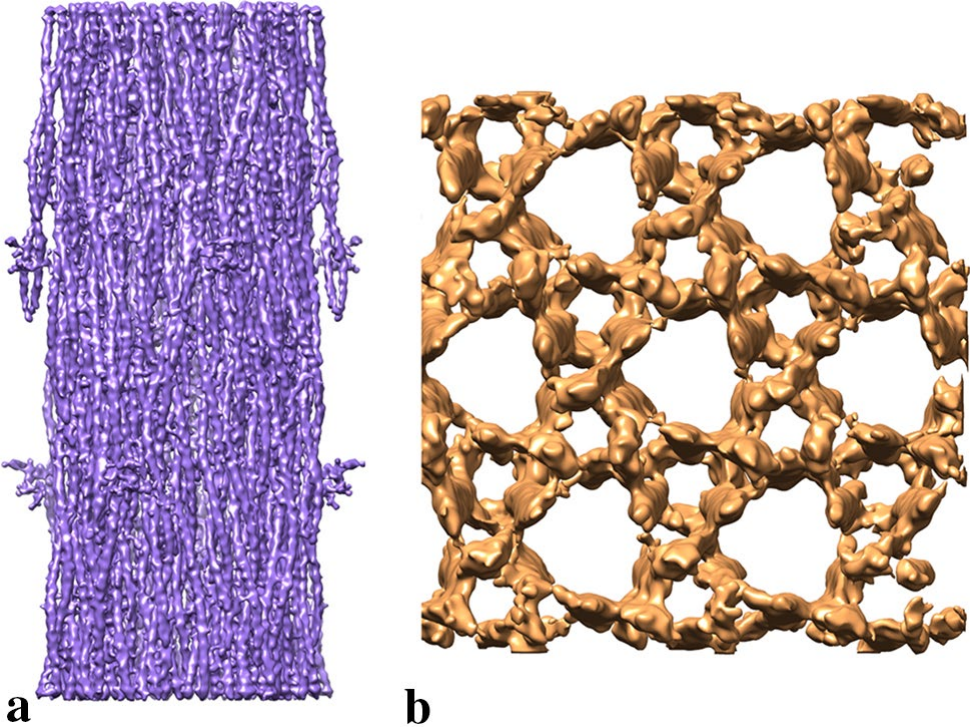
**a** Cryo-EM structure of the relaxed thick filament from Lethocerus flight muscle (EMD-3301, 5.5 Å) (Hu et al. 2016). **b** Cryo-ET structure of frozen-hydrated honeybee Z-disk (EMD-8727, 60.0 Å) (Rusu et al. 2017). Map comparisons not to scale

Since the use of direct electron detectors, the highest reported resolution for a thick filament structure was from Lethocerus at 6 Å shown in Hu et al. 2016 (Fig. 3a). The map shows unprecedented detail in the backbone with α-helical coiled coil myosin rod domains being resolved and shown to lie in a layered arrangement as described in Squire (1973). The myosin heads which sit out at a higher radius were recovered at a much lower resolution of ~ 20 Å, predominantly due to their flexible nature. The heads were found to lie in a similar arrangement to other muscles, with an intramolecular head–head interaction rather than the intermolecular one predicted. This work also gave an insight into the mechanism of stretch activation through the coupling of myosin heads to the backbone.

## Sarcomere structure and cryo electron tomography

Whilst much can be learnt from looking at the 3D structure of isolated muscle filaments, their interactions and arrangement in situ, in the sarcomere also provide important information about muscle function. McDowall et al. (1984) looked at frozen hydrated sections of insect flight muscle as a way of establishing whether the rapid freezing technique they were pioneering faithfully recovered the native structure of the sarcomere. Optical diffractograms of these frozen hydrated sections were compared to previous X-ray diffraction patterns. They concluded that the vitrified specimen was well preserved, avoiding preparation artefacts due to conventional plastic sectioning. This work emphasised the importance of good vitrification to preserve order and avoid the presence of crystalline ice which disrupts the molecular structure, with cubic ice making myosin filaments appear hollow.

Dubochet’s early analysis was a 2D analysis of sarcomere structure. To calculate the 3D structure electron tomography is commonly used, where a series of images at different tilts are recorded. One of the most recent Cryo-EM structures of part of the sarcomere was from Rusu et al. (2017) (Fig. 3b). Z-disks isolated from the honeybee flight muscle were studied using electron tomography, due to their thin structure of ~ 120 nm, sectioning could be avoided. The data were collected on a 300 kV electron microscope with a direct electron detector. A reconstruction at ~ 60 Å resolution was calculated and subtomogram averaging techniques were used to investigate the arrangement of proteins in the hexagonal lattice. Density attributed to α-actinin was visible and density from the actin filaments was improved after subtomogram averaging. Whilst successfully demonstrating the potential of studying vitrified isolated Z-disks, the resolution was limited due to the harsh isolation procedure.

Cryo-electron microscopy of vitrified sections or CEMOVIS requires the ability to section frozen tissue directly. This technique first developed in the 1970s, was driven forward by the commercial development of cryo-ultramicrotomes. There are however known artefacts like crevasses and compression that occur with this particularly difficult technique of acquiring vitrified sections (Al-Amoudi et al. 2004, 2005).

Recently a new approach has been employed to cut thin sections or lamella from vitrified cells and tissue. Focussed ion beam (FIB) milling uses a beam of heavy ions to mill/thin regions of a vitrified sample that is already mounted on an EM grid (Rigort et al. 2012). The grid can then be directly used for Cryo-ET. This exciting methodological development was demonstrated by Wagenknecht et al. (2015) with FIB-milled high-pressure frozen toad fish swim bladder muscle. They were able to visualise T-tubules, triad junctions and individual Ryanodine receptors.

## The future of Cryo-EM in muscle research

Advances in EM technology and particularly camera design are still happening at a rapid pace. There is potential for bigger and faster, electron counting sensors which would result in quicker recording and higher quality images. By capturing more molecular images and profiting from the power of advanced image processing techniques, different functional states will become easier to visualise. Advances in EM go hand in hand with computational advances and as the amount of data being produced on the microscope can be tens of TB, it becomes a data science challenge. Large amounts of data can prove difficult to store and process, but the statistical power it provides will aid maximum likelihood analysis methodology. The development of machine learning and deep learning classifiers for particle detection and classification will also be crucial in the analysis of such large datasets. Accessibility is increasing, not only to the substantial CPU and/or GPU compute power needed to run Cryo-EM software, but the packages themselves are now being designed for non-expert users.

As mentioned above, FIB-milling of samples for Cryo-ET is an exciting prospect for looking at sarcomere structure. The development of this technology further will allow vitrified, well-preserved thin sections devoid of cutting artefacts to be imaged directly in a high resolution Cryo-EM. Rapid freezing without stain should make visualising many if not all the active states of muscle possible. Sarcomeric proteins are also excellent targets for Cryo-ET and subtomogram averaging due to their periodic arrangements. EM has always played an important part of muscle research and it continues to be at the forefront of molecular understanding of function. As resolution increases, visualising the effect of disease-causing mutations and the effect of therapeutics is truly within our grasp.
